# Ultrasonography and the Reorientation of Developmental Hip Dysplasia Research: An NLP-NMF Trend Analysis (1980-2024)

**DOI:** 10.5152/eurasianjmed.2026.251249

**Published:** 2026-03-17

**Authors:** Recep Taşkin, Sinan Yılar, İbrahim Budak, Fatih Uğur

**Affiliations:** 1 Orthopedics and Traumatology Department, Kastamonu University Faculty of Medicine, Kastamonu, Türkiye; 2 Orthopedics and Traumatology Department, Atatürk University Faculty of Medicine, Erzurum, Türkiye; 3 Data Analysis Monitoring and Evaluation Office, Rectorate, Kastamonu University, Kastamonu, Türkiye

**Keywords:** Developmental dysplasia of the hip, Graf classification, natural language processing, non-negative matrix factorization, segmented regression, ultrasonography

## Abstract

**Background:**

Graf’s introduction of hip ultrasonography (US) in the 1980s coincided with a major change in developmental hip dysplasia (DDH) screening and diagnostic practice, with a gradual reduction in reliance on examination- and radiograph-only strategies.

**Methods:**

A natural language processing pipeline and non-negative matrix factorization (NLP–NMF) were applied to Scopus-indexed DDH publications, extracting topics and weights across 4 epochs (pre-1980, 1980-1984, 1985-1989, 1980-2024) anchored to the 1980 US milestone. Topic co-occurrence networks, weight distributions, and segmented regression summarized thematic shifts and tested for a 1980 breakpoint.

**Results:**

In the post-1980 corpus, a distinct diagnostic imaging theme emerged and strengthened, while surgery declined and anatomy remained influential. In 1985-1989, joint anatomy dominated (39.1%), followed by diagnosis (18.6%), pediatric orthopedics (15.3%), surgery (13.7%), and conservative treatment (13.4%). Across 1980-2024, clinical evaluation accounted for 31.5% of publications, followed by anatomy (19.6%), genetic anomaly (16.7%), diagnostic imaging (16.6%), and surgery (15.6%). Within diagnostic imaging, US carried a high weight (0.948), underscoring its central role. Segmented regression identified a structural breakpoint around 1980, temporally coincident with the period in which hip US entered clinical practice and diffused across the field.

**Conclusion:**

The NLP–NMF analysis of 4 decades of DDH literature shows a sustained pivot toward imaging-supported, clinically anchored evaluation and growing attention to etiologic and syndromic/anomaly contexts alongside anatomical determinants, with potential implications for earlier detection, risk-aligned follow-up, and more targeted use of operative management.

**Main Points**

- A natural language processing pipeline and non-negative matrix factorization analysis of 4 decades of developmental hip dysplasia (DDH) literature identifies a structural breakpoint around 1980, with a sustained pivot from surgery-centered themes toward diagnostic imaging and clinically anchored evaluation.
- Across 1980-2024, clinical evaluation (31.5%) and anatomy (19.6%) remained the dominant themes, while surgery became the least prominent (15.6%), consistent with an increased emphasis on earlier, imaging-supported evaluation and a relative de-emphasis of surgery-focused themes in the literature.
- A distinct diagnostic imaging theme emerged post-1980, with “ultrasonography” showing a very high keyword weight (0.948) and central network position, underscoring ultrasonography as a bridging modality between clinical, anatomical, and surgical research domains.
- The growing weight of the genetic-anomaly and anatomy themes suggests increasing attention to etiologic questions and syndromic/anomaly contexts (including familial risk) alongside structural determinants, rather than a direct rise in genomic DDH research.

## Introduction

Developmental hip dysplasia (DDH) comprises a spectrum of disorders involving abnormal development of the acetabulum, proximal femur, and femoral head in infants and young children. If inadequately treated, DDH may lead to substantial functional impairment and is a major cause of early-onset osteoarthritis and hip replacement in young adults.^1^ Clinical presentation ranges from mild acetabular dysplasia to complete hip dislocation, and delayed diagnosis reduces the effectiveness of available treatment options, underscoring the importance of early detection and intervention.^2^

The most consistent risk factors for DDH include family history, breech presentation, and female sex. A recent high-level meta-analysis demonstrated that family history and breech presentation exert the strongest effects in cases confirmed by ultrasonography (US) within the first 3 months of life and that oligohydramnios is also associated with a significantly increased risk. These findings refine the classic risk profile (family history/female sex/breech presentation) reported in previous systematic reviews and help justify referral criteria within screening algorithms.^3^

Graf’s introduction of hip US in 1980 marked an inflection point in the diagnostic approach to DDH and was followed by a wide uptake of ultrasonography-based assessment in many settings.^4^ Ultrasonography has since become the gold standard technique for young infants, providing high sensitivity and specificity for early detection.^5^ Before its adoption, physical examination maneuvers such as Ortolani and Barlow, together with radiographic imaging, were the main diagnostic tools, but these methods were subjective and less sensitive in early infancy.^6^ Ultrasonography is non-invasive, radiation-free, and particularly effective in visualizing cartilaginous structures, monitoring acetabular development, and assessing treatment response through dynamic imaging.^6^^,^^7^ Consequently, hip US has been integrated into newborn screening programs in many countries, facilitating earlier diagnosis, timely initiation of conservative treatment, and a reduction in surgical interventions and long-term complications, although debate persists regarding universal vs. selective screening strategies.^8^

Universal US screening clearly increases early detection compared with selective screening, yet large-scale and multicenter data show no consistent differences between strategies in late diagnosis or operation rates; however, delayed referrals appear less frequent under universal protocols in single-center cohorts. Current practice guidelines often advocate layered algorithms, such as US at 6 weeks followed by a single anteroposterior pelvic radiograph at 6 months, particularly for breech births.^9^ The 4- to 6-month window is critical for post-screening confirmation: American Academy of Orthopaedic Surgeons (AAOS) evidence-based guidelines suggest that radiography may be prioritized from the fourth month onward, whereas the 2022 criteria emphasize a “US at 6 weeks + single Anteroposterior (AP) pelvic radiograph at 6 months” pathway for infants with breech presentation. This practical distinction supports the clinical relevance of modality-based annual publication rates and the post-1980 breakpoint analysis in the study.^10^

Against this background, it is important to characterize how diagnostic and therapeutic emphases in the DDH literature have evolved, especially before and after Graf’s landmark US publication. This study therefore investigates shifts in research themes using non-negative matrix factorization (NMF). The study aimed to delineate how clinical practice and treatment outcomes have been represented over time and to link publication trends with key advances in diagnostic and treatment approaches.

Within this framework, natural language processing (NLP) is employed as a core artificial intelligence (AI) method for transforming free text into analyzable data, enabling automated extraction, coding, and semantic interpretation of documents such as clinical notes and radiology reports.^11^ In the present study, a large DDH literature corpus was processed using NLP techniques combined with NMF to identify terms, frequencies, and contextual patterns that reflect diagnostic and treatment trends. Compared with probabilistic topic models such as LDA and more recent transformer-based approaches like BERTopic, NMF offers fragmented yet interpretable components in the word–document matrix and facilitates clinically meaningful visualization of co-occurrence clusters, with robust algorithms available for near-separable scenarios. This transparency and ease of interpretation motivated the preference for NMF.

Reproducible text-mining research requires sharing of code, queries, and abstract-level data. The FAIR principles (Findable, Accessible, Interoperable, Reusable) promote machine-based discovery and reuse of data and workflows, yet evidence suggests that transparency practices in biomedical literature remain suboptimal.^12^ In this context, the study plans to share Scopus query strings, cleaning procedures, and NMF hyperparameters alongside the code and abstract data.

Based on these considerations, it was hypothesized that the thematic evolution of DDH research exhibits a temporal breakpoint around the period when US became established, with increasing emphasis on diagnostic imaging and etiology-focused themes. Furthermore, it was posited that AI-based approaches such as NLP and NMF can reliably detect these temporal shifts and that future research will increasingly emphasize genetic and anatomical determinants rather than surgical treatment.

## Material and Methods

**Figure 1.**
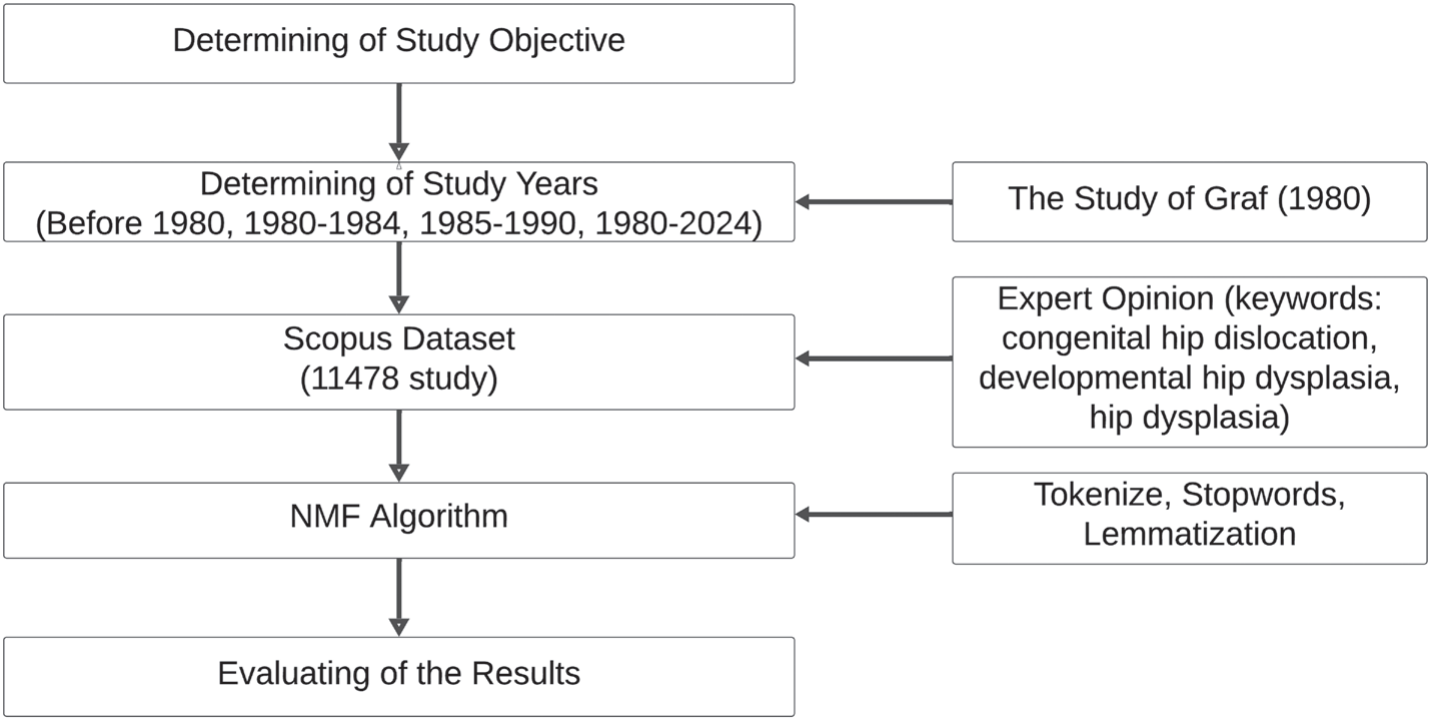
The Methodological Framework of the Study.

This section summarizes the non-negative matrix factorization (NMF/NNMF) approach used in this study (Figure 1). Scopus-indexed articles on DDH constituted the study corpus; US’s introduction in 1980 was treated as a critical milestone, and publications were analyzed for the periods pre-1980 and 1980-2024, with the latter further subdivided into 1980-1984 and 1985-1989 to capture early post-introduction trends.^4^ Using NMF, keyword weights were estimated across the corpus and grouped into 5 themes, which were subsequently labeled based on their constituent terms.

The search universe was defined within Scopus according to Preferred Reporting Items for Systematic Reviews and Meta-Analyses (PRISMA-S) recommendations. Given Scopus’ strong bibliographic coverage, Google Scholar back-checking and reference chaining were additionally applied to minimize selection bias.^13^ Title–abstract texts were lowercased, stripped of punctuation and numbers, and processed with a scientific/biomedical stop list. Bigram–trigram candidates were identified using punctuation and conjunction filters, and scispaCy models were used for lemma normalization, reflecting their proven performance in clinical–scientific texts.^14^

A 1-3-gram TF–IDF term–document matrix was constructed (document frequency ≥2, sublinear TF, L2 normalization), consistent with classical information retrieval evidence supporting TF–IDF for topic discrimination.^15^ The number of topics (K) was selected by scanning k = 5-20 and assessing stability in 5-fold document subsets using C_v and NPMI coherence metrics; both the peak and shoulder regions were considered, given the advantages of C_v/NPMI in capturing semantic alignment.^16^ Based on coherence and stability, K = 5 was selected as the most parsimonious solution that preserved interpretability and minimized conceptual overlap across themes.

For naming and validation, the top 20 terms and 15 highest-weighted documents per topic were reviewed, and theme labels were independently assigned by 2 researchers; disagreements were resolved by consensus, and inter-rater reliability was quantified using Cohen’s kappa statistic (*κ* = 0.73).^17^

### Operational Definitions of the Five NMF Themes

Clinical evaluation: Clinical examination, screening pathways, risk stratification, follow-up algorithms, and clinical scoring/assessment terms (e.g., exam, screening, risk, infant/newborn, clinical, score).

Diagnostic imaging: Imaging modalities and imaging-based diagnosis/monitoring terms (e.g., US, radiograph/X-ray, tomography, imaging).

Diagnosis–treatment (pre-1980 theme): Combined diagnostic and management language in earlier abstracts where diagnostic workup and treatment are not clearly separable as standalone themes.

Anatomy: Anatomical structures, morphology, acetabular/femoral geometry, measurements/angles, and structural descriptors.

Surgery: Operative techniques, osteotomy/reduction, perioperative terms, and postoperative/complication language.

### Ethics

This study was conducted using only open-access Scopus data and did not involve any human or animal experiments; therefore, it did not require ethical committee approval.

### Non-Negative Matrix Factorization

Artificial intelligence (AI) in healthcare offers opportunities to improve outcomes, reduce costs, and support public health.^18^ In DDH, AI is particularly useful for analyzing diagnostic and treatment trends. Two main branches are relevant: machine learning, which uses genetic and imaging data to predict outcomes or cluster patients, and NLP, which extracts information from unstructured text and accelerates literature analysis by grouping word patterns in large corpora.^19^

Non-negative matrix factorization is a widely used method for dimensionality reduction and data representation in text mining, image processing, and bioinformatics. It decomposes a non-negative data matrix into 2 lower-rank non-negative matrices, enabling the extraction of latent features and supporting clustering, classification, and feature extraction.^20^^,^^21^ The sparse, interpretable representations produced by NMF are advantageous in complex data domains, and recent extensions—such as sparsity constraints and alternative similarity measures—have further enhanced its applicability.^21^

In NLP and text mining, NMF transforms a sparse term–document matrix into a lower-dimensional space that captures underlying semantic structure and is used for document clustering, feature extraction, and classification. For example, Barman et al applied a 2-stage NMF approach to the Classic3 dataset and achieved >98.5% classification accuracy by first extracting basis vectors and then using them for clustering or classification.^22^ Compared with PCA or ICA, NMF’s aggregation-based, parts-based representations are often more intuitive for non-negative data, improving interpretability.^20^ Optimization is typically performed with iterative multiplicative update rules, such as those proposed by Lee and Seung, which preserve non-negativity while minimizing reconstruction error.^23^^,^^21^

For the term co-occurrence network, a window-based transition matrix was constructed, normalized using association strength, and thresholded to retain meaningful edges. Network mapping and clustering were performed with the VOS technique, and degree and betweenness centralities were reported.^24^ Segmented regression modeling incorporated 1980 as the breakpoint year, with level and slope change parameters; pre-intervention trend continuity and autocorrelation were addressed using Newey–West HAC robust errors and Prais–Winsten AR(1) FGLS, with Durbin–Watson and Ljung–Box statistics for diagnostics.^25^ In segmented regression, the dependent variable was the annual theme share (i.e., the proportion of total theme weight attributable to each theme in a given year). Sensitivity analyses varied the breakpoint (1978-1982), preprocessing options (stop-lists, n-gram limits), topic number around the coherence peak (±1), and alternative topic models.

All analyses were conducted in Python using scikit-learn for NMF and TF–IDF, statsmodels for interrupted time-series regression, and gensim for n-gram processing and utility functions.

## Results

**Figure 2.**
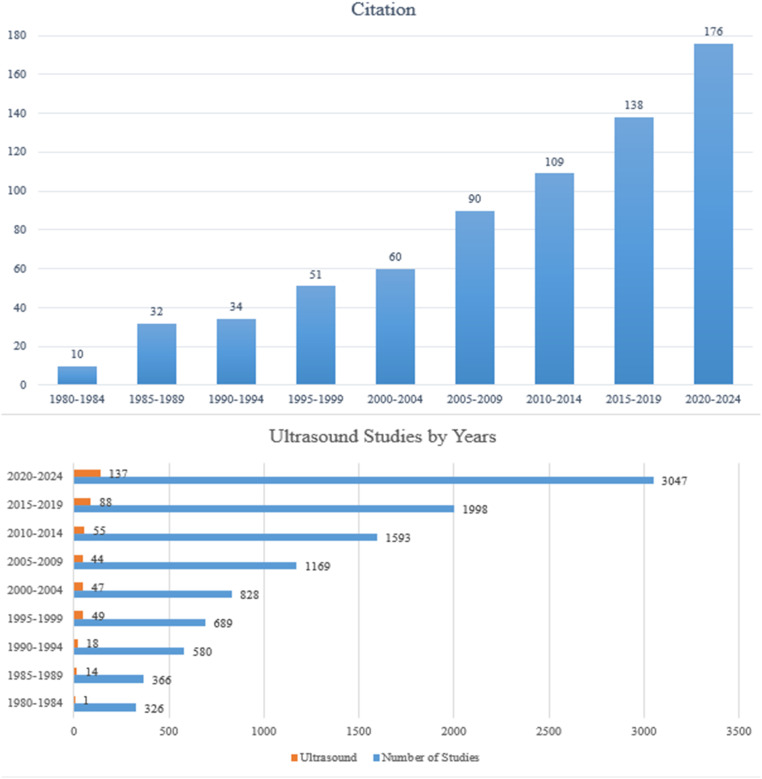
Annual Number of Publications and Citations in the DDH Literature (Scopus).

The Scopus corpus was analyzed with the NMF algorithm, and the most frequent keywords were grouped into clusters. Each theme was titled by the researchers based on its dominant terms. Because US represents a major milestone in DDH diagnosis, the annual number of publications and citations in Scopus was also examined (Figure 2). Between 1980 and 1984, only 10 studies cited Graf’s original US paper, 4 of which were authored by Graf himself.

Segmented regression with 1980 as the breakpoint showed significant level (β_2_) and slope (β_3_) changes in theme proportions. The imaging-focused share exhibited an immediate increase followed by a positive slope change, whereas the surgery-focused share showed a declining slope (imaging: β_2_ = 0.14, 95% CI 0.07-0.21, *P* = .001; β_3_ = 0.012, 95% CI 0.006-0.019, *P* = .002; surgery: β_2_ = −0.06, 95% CI −0.14-0.03, *P* = 0.17; β_3_ = −0.009, 95% CI −0.014 to −0.004, *P* = .003). This pattern aligns with recommended approaches for separating short- and long-term level and slope changes of practice changes in time-series patterns.

Here, β_2_ represents the immediate change in theme share at the 1980 breakpoint (percentage-point change), whereas β_3_ represents the additional annual change in theme share after 1980 relative to the pre-1980 trend. For example, β_2_ = 0.14 indicates an immediate ~14 percentage-point increase in the imaging theme share at 1980, and β_3_ = 0.012 indicates an additional ~1.2 percentage-point increase per year thereafter.

In the pre-ultrasonography era, NMF identified themes related to joint, genetics, diagnosis–treatment, surgery, and rehabilitation. Articles published before 1980 focused mainly on diagnosis–treatment (27.6%), followed by joint and surgery (22.3%), rehabilitation (14.8%), and genetics (12.9%) (Table 1). The dominance of diagnosis–treatment and joint–surgery themes reflects reliance on clinical examination and radiography, both of which have limited diagnostic accuracy in early infancy.

The early US period was examined in 2 subintervals. From 1980 to 1984, themes included joint diseases, hip problems, joint–cartilage health, surgery, and anatomy–anomaly. Publications were mainly on joint diseases (40.5%), followed by hip problems (22.1%), surgery (14.7%), joint–cartilage health (13.5%), and anatomy–anomaly (9.2%) (Table 2). Between 1985 and 1989, themes shifted to joint anatomy, pediatric orthopedics, treatment, diagnosis, and surgery, with articles predominantly addressing joint anatomy (39.1%), then diagnosis (18.6%), pediatric orthopedics (15.3%), surgery (13.7%), and conservative treatment (13.4%) (Table 3). The rising emphasis on anatomy and the modest decline in surgery (≈13.7% in 1985-1989) parallel the move toward systematic screening and earlier intervention, consistent with reports of reduced late diagnosis and operation rates in several national programs.

For the entire 1980-2024 period, the thematic structure comprised clinical evaluation, diagnostic imaging, genetic anomaly, anatomy, and surgery. Publications most frequently addressed clinical evaluation (31.5%), followed by anatomy (19.6%), genetic anomaly (16.7%), diagnostic imaging (16.6%), and surgery (15.6%) (Table 4). Clinical evaluation and anatomy remained prominent, while the high weight of the keyword US within the diagnostic imaging theme (0.948) underscored its bridging role in the network. High degree and especially betweenness centrality of imaging-related nodes in the co-occurrence graph indicate that imaging functions as an intermediary in the flow of information between topics. Although “clinical evaluation” and “diagnostic imaging” are clinically related, in the NMF solution they separate operationally: the former captures exam/screening/risk/follow-up language, whereas the latter concentrates modality-specific imaging terminology (e.g., US/radiography/tomography).

The thematic profile aligns with the literature on the performance of the Graf method and the limited sensitivity of clinical examination alone. Meta-analyses of Graf US report high sensitivity and specificity, whereas clinical-exam-based screening shows substantial variability. Recent gains in the accuracy of AI-assisted imaging (e.g., automated Graf-type classification and key-point detection) further strengthen the centrality of the imaging theme.

The overall thematic structure remained stable across C_v coherence peaks in the range *k* ∈ (5-20). Sensitivity analyses using consensus/cophenetic stability and alternative topic models confirmed the persistence of the main themes: clinical evaluation, anatomy, imaging, genetic–anomaly, and surgery.

Because Scopus’ pre-1980 indexing is less complete than that of WoS and PubMed/MEDLINE, the pre-1980 thematic distribution should be interpreted cautiously as a Scopus-indexed subset and may undercapture early European, non-English, and surgically oriented reports.

## Discussion

The key observation of this study is that the DDH literature shows a durable thematic reorientation temporally aligned with the post-1980 period, during which hip US became widely adopted. A distinct “diagnostic imaging” theme emerged and strengthened over time, accompanied by rising weights for US-related terms and a relative decline in surgery-related themes, whereas “anatomy” remained influential across all periods. These temporal patterns suggest that imaging-supported clinical evaluation has become increasingly prominent within the literature and suggest that future work will prioritize etiologic and syndromic/anomaly contexts (including familial risk) alongside anatomical determinants, rather than a direct rise in genomic research over surgical techniques.

The dominance of the “clinical evaluation” theme likely reflects its broader operational scope in abstracts, which frequently summarize screening, examination, and follow-up decisions alongside diagnostic workup. In addition, clinical evaluation terms co-occur with both imaging and management language, making this theme a high-frequency “hub” in abstract-level reporting.

Regarding screening strategies, the literature reports heterogeneous findings on the impact of universal US in reducing late diagnosis and the need for surgery. A major meta-analysis showed that universal US increases early detection but does not clearly outperform selective US or clinical screening alone in terms of late diagnosis and operative treatment rates, whereas more recent pooled data and national cohorts suggest lower late-DDH incidence under universal screening.^9^ These discrepancies underscore the importance of program design, implementation quality, and follow-up algorithms. From a health-policy perspective, universal US improves coverage and equity but carries higher direct program costs; systematic reviews indicate that overall cost–effectiveness depends on downstream effects on surgery, rehabilitation, long-term care, parental work loss, and inequalities, and must therefore be evaluated in a broad societal framework.^26^

With the rapid growth of health data, AI has become increasingly important in healthcare,^27^ and manual analysis of large text corpora is difficult.^28^ Non-negative matrix factorization, which produces interpretable low-dimensional representations,^21^ enabled efficient analysis of all Scopus-indexed DDH articles. Using keyword weights, themes were identified that both summarize the historical evolution of DDH research and align with prior conceptualizations in the literature.^20^

Before 1980, when US was not yet incorporated into DDH diagnostics, NMF revealed a dominant “diagnosis–treatment” theme, followed by “hip joint” and “surgery” (Table 1). High weights for terms such as “hip,” “luxation,” “infant,” “dysplasia,” and “treatment” emphasize the focus on recognizing and managing disease in early life, including both conservative and surgical approaches. The similar weights of the top 3 themes suggest that surgery played a central role within joint-focused diagnosis and treatment in this era. In 1980-1984, “joint diseases” remained predominant (40.5%), but the relative weight of surgery decreased (14.7%) and fell further to 13.7% between 1985 and 1989. Although a distinct “diagnosis” theme was not detected for 1980-1984, it became the second most prominent theme after “joint anatomy” in 1985-1989 (Table 3). Across the entire post-1980 period, “clinical evaluation” was the leading theme (31.5%), and “diagnostic imaging” appeared as a separate theme (16.6%), indicating that DDH diagnosis increasingly integrated both imaging modalities and clinical examination; the NMF results also show that diagnostic imaging has become analytically separable from both clinical assessment and surgery (Table 4).

A significant proportion of mild dysplasias detected by early US may resolve spontaneously, raising concerns about overtreatment, especially in stable, mild cases. The literature indicates that “delayed US + targeted splinting” protocols can reduce unnecessary treatment without increasing delayed diagnosis or surgery rates, whereas some cohorts still report a reduction in late DDH with universal screening.^29^ This dual pattern highlights the need to calibrate local risk profiles and clinical workflows carefully.

Network metrics in the analysis show that imaging-driven themes have high degree and betweenness centralities, acting as bridges between subfields. Because centrality in weighted networks incorporates both link strength and number of connections, this supports the interpretation that diagnostic imaging themes are both frequently referenced and function as key gateways for information flow, potentially explaining the rapid dissemination of methodological innovations (e.g., US protocols) across conceptual clusters.^30^

Although US was described as a diagnostic method by Graf,^4^ the findings (Table 2) indicate that it was not rapidly adopted in the early 1980s: only 10 studies in that period cited Graf’s paper, 4 authored by Graf himself, and more attention was still given to modalities such as tomography (e.g., weight 0.175 under “anatomy–anomaly”). Between 1985 and 1989, “joint anatomy” became the leading theme (39.1%), followed by “diagnosis” (18.6%), and US emerged as a high-weight keyword, second only to “hip” within the diagnosis theme. Consistent with the literature, US has been reported to have high diagnostic sensitivity in DDH and to provide dynamic visualization during treatment,^6^^,^^7^ which likely drove its broader clinical adoption. In the post-1980 corpus analyzed without subperiods, a distinct “diagnostic imaging” theme was observed (Table 4), and the weight of US within this theme increased markedly (0.948). Graf’s early work thus appears to have stimulated a line of research that expanded over subsequent decades.^4^

This study has several limitations. (i) Reliance on Scopus as the sole data source is a major limitation. Scopus’ historical coverage—particularly for the pre-1980 period—differs from Web of Science and PubMed/MEDLINE and may introduce systematic selection bias (e.g., under-representation of early European, non-English, or surgically oriented literature). Therefore, early-period thematic proportions should be interpreted cautiously as reflecting the Scopus-indexed subset of the literature. Future studies should adopt multi-database retrieval (e.g., Scopus + WoS + PubMed) with deduplication to improve historical completeness and reduce coverage bias. (ii) Abstract-level text mining may diverge from full-text modeling in some topic distributions. (iii) In NMF-based topic modeling, the number of topics and coherence metrics are sensitive to parameter choices, and high coherence does not always guarantee more meaningful topics. Accordingly, the findings should be interpreted alongside sensitivity analyses and alternative model comparisons.^31^ (iv) Although segmented regression can detect temporal breakpoints, the analysis does not support causal inference. Multiple contemporaneous factors (e.g., advances in neonatal care, epidemiologic awareness, surgical techniques, growth in publication volume, and editorial policies) were not modeled and may also have contributed to the observed thematic changes.

An additional notable finding is the behavior of the “anatomy” theme. “Anatomy” was absent as a distinct theme or keyword cluster before 1980 but gained substantial weight afterward. In 1980-1984, it appeared in combination with “anomaly,” likely reflecting increased anatomical comparisons to elucidate congenital anomalies; the disappearance of “anomaly” as a separate term after 1984 may indicate that anomalies became conceptually integrated within anatomical studies rather than treated as distinct entities. By 1985-1989, “joint anatomy” surpassed both diagnostic and therapeutic themes in weight, and anatomical research has maintained its prominence through to the present. In contrast, “surgery” became the least prominent theme in 1980-2024, suggesting that research increasingly prioritized understanding disease mechanisms over describing treatment techniques. Taken together, these patterns support the expectation that future studies will focus more on etiology and syndromic/anomaly contexts (including familial risk) and detailed hip joint anatomy (Table 4).

## Conclusion

Using an NLP-driven NMF analysis of the DDH literature, major thematic shifts were quantitatively identified over time. Before 1980, research emphasized diagnosis and treatment; in the post-1980 period (coincident with the diffusion of hip US into clinical practice), diagnostic imaging emerged as a distinct and influential theme, while surgery steadily declined. Anatomy maintained consistent prominence across periods. These temporal patterns suggest that imaging-supported clinical evaluation has become increasingly prominent within the literature and that future work will likely prioritize etiologic, genetic, and anatomical determinants over surgical techniques. Artificial intelligence–based text mining provides a rapid, reproducible way to reveal such trends and to evaluate the temporal association between diagnostic innovations and thematic trajectories.

## Figures and Tables

**Table 1.** Identification of Themes and Keywords with NMF before 1980

**Hip Joint** **Word**	**22.30%** **Weight**	**Genetics** **Word**	**12.90%** **Weight**	**Diagnosis-Treatment** **Word**	**27.60%** **Weight**	**Surgery** **Word**	**22.30%** **Weight**	**Rehabilitation** **Word**	**14.80%** **Weight**
Acetabular	1.394	Variant	1.140	Hip	0.734	Osteotomy	0.705	Hip	1.240
Femoral	0.856	Gene	0.602	Infant	0.513	Hip	0.548	Laxity	0.778
Angle	0.828	Syndrome	0.522	Luxation	0.491	Femoral	0.500	Labral	0.490
Hip	0.694	Genetic	0.477	Child	0.471	Revision	0.496	Score	0.442
Head	0.609	Mutation	0.408	Treatment	0.448	Crowe	0.463	Pain	0.424
Pelvic	0.536	Pathogenic	0.376	Risk	0.435	Complication	0.418	Tissue	0.317
Coverage	0.479	Disorder	0.361	Subluxation	0.414	Postoperative	0.400	Capsular	0.299
Dysplasia	0.446	Case	0.355	Dysplasia	0.388	Year	0.392	Borderline	0.277
Anterior	0.437	Congenital	0.341	Reduction	0.349	Score	0.318	Level	0.249
Parameter	0.416	Phenotype	0.334	Factor	0.320	Survival	0.312	Improvement	0.246
Measurement	0.412	Sequencing	0.329	Age	0.318	Surgery	0.308	Dysplasia	0.237
Correlation	0.379	Clinical	0.326	Developmental	0.315	Clinical	0.301	Impingement	0.235
Lateral	0.348	Feature	0.316	Diagnosis	0.306	Surgical	0.299	Femoroacetabular	0.225
Dysplastic	0.341	Rare	0.313	Examination	0.270	Iv	0.295	Treatment	0.218
Value	0.337	Skeletal	0.310	Die	0.255	Bone	0.275	Minimum	0.216
Radiograph	0.332	Individual	0.301	Early	0.246	Shortening	0.273	Difference	0.195
Acetabulum	0.327	Associated	0.286	Newborn	0.235	Length	0.273	Athlete	0.191
Normal	0.315	Heterozygous	0.283	Incidence	0.234	Primary	0.262	Significant	0.191
Radiographic	0.313	Identified	0.280	Pediatric	0.218	Case	0.261	Estradiol	0.187
Anteversion	0.313	Novel	0.275	Technique	0.205	Dislocation	0.245	Surgery	0.177

**Table 2.** Identification of Themes and Keywords with NMF in 1980-1984

**Hip Joint ** **Disease** **Word**	**40.5%** **Weight**	**Hip ** **Problem Word**	**22.1%** **Weight**	**Joint-Cartilage ** **Health** **Word**	**13.5%** **Weight**	**Surgery** **Word**	**14.7%** **Weight**	**Anatomy-Anomaly** **Word**	**9.2%** **Weight**
Avascular	0.805	Hip	0.456	Joint	0.738	Osteotomy	0.390	Femoral	0.650
Necrosis	0.736	Treatment	0.445	Hip	0.371	Operation	0.331	Head	0.354
Reduction	0.416	Dislocation	0.350	Dysplasia	0.271	Year	0.329	Acetabulum	0.268
Hip	0.403	Screening	0.288	Cartilage	0.192	Performed	0.287	Acetabular	0.240
Incidence	0.345	Child	0.283	Degenerative	0.176	Procedure	0.281	Dislocation	0.240
Traction	0.338	Infant	0.277	Change	0.176	Excellent	0.260	Congenital	0.225
Treated	0.229	Case	0.263	Disease	0.175	Hip	0.248	Hip	0.221
Femoral	0.224	Congenital	0.245	Breed	0.155	Good	0.244	Anteversion	0.206
Harness	0.218	Neonatal	0.236	Dysplastic	0.154	Poor	0.243	Neck	0.206
Closed	0.191	Newborn	0.230	Normal	0.153	Fair	0.219	Reduction	0.175
Child	0.183	Early	0.226	Collagen	0.144	Age	0.215	Tomography	0.175
Dislocated	0.179	Examination	0.216	Similar	0.118	Obtained	0.183	Osteotomy	0.164
Dislocation	0.179	Diagnosis	0.185	Multiple	0.113	Followed	0.178	Angle	0.156
Development	0.164	Cdh	0.183	Significant	0.111	Derotation	0.166	Proximal	0.145
Contralateral	0.156	Instability	0.159	Articular	0.107	Ten	0.156	Deformity	0.142
Abducted	0.154	Age	0.152	Development	0.106	Five	0.147	Bone	0.141
Review	0.149	Diagnosed	0.142	Observed	0.105	Three	0.145	Position	0.133
Congenitally	0.145	Cent	0.141	Acetabular	0.103	Case	0.143	Case	0.133
Unilateral	0.143	Period	0.137	Synovial	0.102	Average	0.127	Computed	0.129
Treatment	0.134	Baby	0.134	Canine	0.102	Sequela	0.123	Femur	0.117

**Table 3.** Identification of Themes and Keywords with NMF in 1985-1989

**Joint anatomy** **Word**	**39.1%** **Weight**	**Pediatric Orthopedics** **Word**	**15.3%** **Weight**	**Conservative Treatment** **Word**	**13.4%** **Weight**	**Diagnosis** **Word**	**18.6%** **Weight**	**Surgery** **Word**	**13.7%** **Weight**
Acetabular	1.135	Cdh	0.432	Reduction	0.612	Hip	0.633	Osteotomy	0.635
Bone	0.356	Baby	0.377	Child	0.512	Ultrasound	0.496	Hip	0.422
Hip	0.339	Screening	0.296	Necrosis	0.363	Infant	0.368	Operation	0.376
Dysplasia	0.302	Hip	0.282	Avascular	0.360	Examination	0.325	Year	0.301
Acetabulum	0.293	Case	0.272	Treatment	0.336	Diagnosis	0.228	Femoral	0.292
Severe	0.276	Early	0.259	Hip	0.275	Method	0.210	Innominate	0.248
Femoral	0.253	Breech	0.247	Treated	0.263	Dysplasia	0.210	Good	0.241
Lateral	0.232	Congenital	0.231	Pavlik	0.255	Clinical	0.196	Age	0.218
Loosening	0.231	Treatment	0.213	Closed	0.247	Normal	0.171	Performed	0.213
Congenital	0.221	Dislocation	0.211	Harness	0.244	Clinically	0.158	Radiographic	0.207
Dislocation	0.194	Diagnosed	0.209	Femoral	0.229	Cartilaginous	0.153	Procedure	0.198
Roof	0.185	Neonatal	0.209	Traction	0.210	Joint	0.153	Complication	0.177
True	0.170	Incidence	0.180	Head	0.177	Technique	0.153	Average	0.176
Graft	0.169	Late	0.178	Raven	0.175	Sonographic	0.149	Chiari	0.170
Development	0.168	Factor	0.169	New	0.167	Dislocation	0.140	Clinical	0.151
Osteoarthritis	0.167	Examined	0.150	Dislocation	0.155	Newborn	0.137	Angle	0.144
Average	0.163	Detection	0.142	Age	0.137	Structure	0.136	Change	0.128
Replacement	0.163	Born	0.142	Splint	0.130	Ultrasonic	0.135	Excellent	0.123
Secondary	0.161	Risk	0.139	Dislocated	0.118	Evaluation	0.132	Pelvic	0.120
Age	0.157	Found	0.138	Followed	0.115	Imaging	0.124	Coxa	0.118

**Table 4.** Identification of Themes and Keywords with NMF in 1980-2024

**Clinical Evaluation** **Word**	**31.5%** **Weight**	**Diagnostic Imaging** **Word**	**16.6%** **Weight**	**Genetic-Anomaly** **Word**	**16.7%** **Weight**	**Anatomy** **Word**	**19.6%** **Weight**	**Surgery** **Word**	**15.6%** **Weight**
Hip	2.482	Hip	1.236	Syndrome	0.778	Acetabular	1.826	Femoral	1.906
Score	1.145	Ultrasound	0.948	Variant	0.770	Angle	0.956	Osteotomy	1.365
Year	1.093	Screening	0.944	Mutation	0.722	Hip	0.688	Head	0.807
Revision	0.855	Infant	0.939	Gene	0.715	Dysplasia	0.494	Reduction	0.721
Pain	0.672	Treatment	0.768	Genetic	0.631	Pelvic	0.458	Hip	0.528
Level	0.587	Child	0.759	Case	0.603	Coverage	0.426	Dislocation	0.508
Survival	0.565	Dysplasia	0.610	Congenital	0.553	Measurement	0.423	Proximal	0.472
Primary	0.556	Examination	0.586	Disorder	0.552	Dysplastic	0.408	Surgical	0.388
Surgery	0.555	Risk	0.586	Disease	0.459	Joint	0.367	Shortening	0.371
Arthroscopy	0.550	Developmental	0.510	Associated	0.452	Acetabulum	0.354	Femur	0.318
Clinical	0.546	Diagnosis	0.460	Clinical	0.415	Radiograph	0.354	Treatment	0.318
Postoperative	0.493	Newborn	0.438	Feature	0.406	Normal	0.354	Avascular	0.314
Age	0.481	Early	0.420	Rare	0.399	Radiographic	0.342	Neck	0.314
Osteoarthritis	0.474	Pavlik	0.418	Skeletal	0.386	Lateral	0.330	Case	0.310
Joint	0.434	Factor	0.393	Present	0.352	Anterior	0.319	Procedure	0.303
Average	0.429	Dislocation	0.385	Phenotype	0.341	Parameter	0.316	Necrosis	0.301
Radiographic	0.420	Harness	0.380	Family	0.324	Correlation	0.306	Technique	0.300
Underwent	0.417	Reduction	0.370	Individual	0.313	Cartilage	0.306	Pelvic	0.289
Dysplasia	0.394	Age	0.365	Characterized	0.307	Value	0.304	Complication	0.288
Loosening	0.391	Clinical	0.350	Identified	0.298	Measured	0.283	Average	0.272

## Data Availability

The data that support the findings of this study are available on request from the corresponding author.
